# Survey data on organizational culture and entrepreneurial orientation in German family firms

**DOI:** 10.1016/j.dib.2019.103827

**Published:** 2019-03-28

**Authors:** Christopher Arz, Andreas Kuckertz

**Affiliations:** Institute of Marketing and Management, Entrepreneurship (570 C), University of Hohenheim, Wollgrasweg 49, 70599 Stuttgart, Germany

## Abstract

This data article describes a dataset of 208 cases representing assessments of entrepreneurial orientation and organizational culture variables obtained from a web-based survey addressing owners and/or CEOs of German family firms. It includes data on five dimensions of entrepreneurial orientation (innovativeness, proactiveness, risk taking, autonomy, competitive aggressiveness), three dimensions of long-term orientation (futurity, perseverance, continuity), six dimensions of stewardship climate (organizational identification, collectivist orientation, power distance, involvement orientation, use of personal power, intrinsic motivation), three dimensions of learning orientation (commitment to learning, shared vision, open-mindedness), willingness to change, error management culture, and family commitment culture. Additionally, firm-level attributes (e.g., industry, age, size) and top management-level characteristics (e.g., generational involvement, involvement of the founder) are included.

**Table:** Specifications table

Subject area	*Strategy and Management*
More specific subject area	*Family Firms, Entrepreneurial Orientation, Organizational Culture*
Type of data	*Comma-separated Values (CSV) (.csv) and Statistical Package for the Social Sciences (SPSS) (.sav)*
How data was acquired	*Web-based survey*
Data format	*Raw, filtered, and partially analyzed*
Experimental factors	*Raw data obtained from a web-based survey addressing owners and CEOs of German family firms; incomplete cases are eliminated.*
Experimental features	*Measures include entrepreneurial orientation, family commitment culture, long-term orientation, stewardship climate, learning orientation, willingness to change, and error management culture.*
Data source location	*Germany*
Data accessibility	*https://data.mendeley.com/datasets/y32vp8tgdg/1*
Related research article	*not applicable*

**Table:** 

**Value of the data** • The dataset includes entrepreneurial orientation and organizational culture variables in family firms that may be examined using statistical methods such as linear regression, factor analysis, or structural equation modeling. • The data on entrepreneurial orientation in family firms allows for comparisons with other studies in the field and may inform potential meta-analyses. • The dataset includes firm-level descriptive attributes such as industry, age, size, and prior performance as well as top management-level characteristics such as generational involvement, involvement of the founder, and CEO tenure. These fields may allow for comparisons of between-group differences from this sample to parallel samples in other similar studies elsewhere.

## 1 Data

Understanding family firms is an important area of investigation in management research and economics [1]. The German economy is a suitable setting to analyze such businesses, given that it is largely shaped by small and medium sized enterprises (“German Mittelstand”), which are quite often family owned. Equally, many larger corporations headquartered in Germany are controlled by families as well. These firms are often said to be characterized by a specific organizational culture determined by the family in control – a phenomenon, which has not yet been sufficiently understood and which allows for a plethora of research opportunities [2].

Against this background, researchers collected data to illustrate organizational culture and entrepreneurial orientation in German family firms. To aid data collection, a family firm is defined as

• a privately held firm where ownership resides within one family,
• where this family is represented in the management team and substantially influences the key decisions and direction of the firm, and
• the business is perceived to be a family firm by the leading representative of the firm.

The dataset contains self-reported responses of individual study participants. Table 1 summarizes the variables in the dataset.^1^ All variables represent either family-, firm- or top management-level concepts or attributes that have been assessed by the owner and/or CEO of a particular German family firm (key informant approach).

**Table 1:** Variables, variable types, type of questions, value labels, and 2nd order constructs.

Field(s)	Variable(s)	Variable type	Type of question	Value labels	2^nd^ order construct
INN1…INN3^a^	Reported position between two polar adjectives of three innovativeness items	Ordinal	5-point semantic differential	–	Entrepreneurial orientation
PRO1…PRO3^a^	Reported position between two polar adjectives of three proactiveness items	Ordinal	5-point semantic differential	–	Entrepreneurial orientation
RIS1…RIS3^a^	Reported position between two polar adjectives of three risk taking items	Ordinal	5-point semantic differential	–	Entrepreneurial orientation
AUT1 … AUT6^a^	Reported agreement with six autonomy items	Ordinal	5-point Likert scale	1: not at all – 5: to an extreme extent	Entrepreneurial orientation
COMP1…COMP2^a^	Reported position between two polar adjectives of two competitive aggressiveness items	Ordinal	5-point semantic differential	–	Entrepreneurial orientation
FCC1...FCC10^a^	Reported agreement with ten family commitment culture items	Ordinal	5-point Likert scale	1: not at all – 5: to an extreme extent	–
CONT1…CONT4^a^	Reported agreement with four continuity items	Ordinal	5-point Likert scale	1: not at all – 5: to an extreme extent	Long-term orientation
FUT1…FUT5^a^	Reported agreement with five futurity items	Ordinal	5-point Likert scale	1: not at all – 5: to an extreme extent	Long-term orientation
PER1…PER3^a^	Reported agreement with three perseverance items	Ordinal	5-point Likert scale	1: not at all – 5: to an extreme extent	Long-term orientation
OI1…OI3^a^	Reported agreement with three organizational identification items	Ordinal	5-point Likert scale	1: not at all – 5: to an extreme extent	Stewardship climate
COLL1…COLL3^a^	Reported agreement with three collectivist orientation items	Ordinal	5-point Likert scale	1: not at all – 5: to an extreme extent	Stewardship climate
LPD1…LPD3^a^	Reported agreement with three power distance items	Ordinal	5-point Likert scale	1: not at all – 5: to an extreme extent	Stewardship climate
IO1…IO3^a^	Reported agreement with three involvement orientation items	Ordinal	5-point Likert scale	1: not at all – 5: to an extreme extent	Stewardship climate
UPP1…UPP3^a^	Reported agreement with three use of personal power items	Ordinal	5-point Likert scale	1: not at all – 5: to an extreme extent	Stewardship climate
IM1…IM3^a^	Reported agreement with three intrinsic motivation items	Ordinal	5-point Likert scale	1: not at all – 5: to an extreme extent	Stewardship climate
CTL1 … CTL3^a^	Reported agreement with three commitment to learning items	Ordinal	5-point Likert scale	1: not at all – 5: to an extreme extent	Learning orientation
SV1…SV4^a^	Reported agreement with four shared vision items	Ordinal	5-point Likert scale	1: not at all – 5: to an extreme extent	Learning orientation
OM1…OM3^a^	Reported agreement with three open-mindedness items	Ordinal	5-point Likert scale	1: not at all – 5: to an extreme extent	Learning orientation
WTC1…WTC4^a^	Reported agreement with four willingness to change items	Ordinal	5-point Likert scale	1: not at all – 5: to an extreme extent	–
EMC1…EMC15^a^	Reported agreement with 15 error management culture items	Ordinal	5-point Likert scale	1: not at all – 5: to an extreme extent	–
FB1	Family ownership	Nominal	Single-choice question	1: yes; 2: no	–
FB2	Perception of firm as being a family business	Nominal	Single-choice question	1: yes; 2: no	–
GI	Generations involved in the family firm	Nominal	Single-choice question	1: One generation, 2: two generations, 3: multiple generations (more than two)	–
IF	Involvement of the founder	Nominal	Single-choice question	1: yes; 2: no	–
Control_role	Role of respondent in the firm	Nominal	Single-choice question	1: owner, 2: owner and CEO, 3: CEO	–
Control_industry	Industry of the firm	Nominal	Single-choice question	1: Automotive, 2: Real Estate, 3: Bio/Medical Technology, 4: Electronics Industry, 5: Chemicals/Pharmaceuticals, 6: Energy/Resources, 7: Financial Services, 8: Trade, 9: IT/Software/Internet, 10: Engineering, 11: Media, 12: Professional Services, 13: Telecommunications, 14: Transport/Logistics, 15: FMCG/food, 16: Processing, 17: Tourism, 18: Health Care, 19: Textile Industry, 20: Other Services, 21: Craft	–
Control_CEO tenure	Tenure of current CEO	Ratio	Open question	Years	–
Control_age	Age of the firm	Ratio	Open question	Year of founding	
Control_size	Total number of employees relative to competitors	Ordinal	Classification question	1: bottom 20%, 2: next lowest 20%, 3: middle 20%, 4: next highest 20%, 5: top 20%	–
Control_prior performance 1	Total sales growth over the most recent year compared to industry competitors	Ordinal	Classification question	1: bottom 20%, 2: next lowest 20%, 3: middle 20%, 4: next highest 20%, 5: top 20%	–
Control_prior performance 2	After-tax return on sales over the most recent year compared to industry competitors	Ordinal	Classification question	1: bottom 20%, 2: next lowest 20%, 3: middle 20%, 4: next highest 20%, 5: top 20%	–

a For the inventory of each of the available items, refer to supplementary material.

Table 2 Contains the descriptive statistics of the variables in the data set and reports the results of appropriate validity and reliability tests.

**Table 2:** Descriptive statistics, validity, and reliability tests.

	Mean	SD	Cr. α	AVE	CR	Item-to-total correlation	Factor loading (EFA)	Factor loading (CFA)
Innovativeness	2.94	1.10	.81	.60	.82			
INN1	2.67	1.32				.56	.78	.64
INN2	3.23	1.29				.70	.88	.85
INN3	2.92	1.29				.71	.88	.82
Proactiveness	3.58	.87	.80	.57	.80			
PRO1	3.64	1.00				.63	.84	.70
PRO2	3.41	1.08				.68	.87	.82
PRO3	3.69	1.02				.62	.83	.74
Risk taking	2.94	.84	.79	.55	.79			
RISK1	2.87	.96				.60	.82	.68
RISK2	3.08	1.01				.62	.83	.77
RISK3	2.88	1.04				.66	.86	.78
Competitive aggressiveness	2.82	.57	.64	.57	.71			
COMP1	2.90	1.03				.47	.86	.50
COMP2	2.74	1.07				.47	.86	.95
Autonomy	4.15	.57	.82	.44	.82			
AUT1	4.14	.77				.62	.76	.68
AUT2	4.08	.79				.63	.77	.71
AUT3	3.69	.92				.62	.76	.71
AUT4	4.36	.71				.54	.69	.60
AUT5	4.13	.82				.67	.80	.76
AUT6	4.50	.70				.39	.53	.46
Continuity	4.24	.58	.67	.41	.73			
CONT1	4.43	.71				.30	.53	.47
CONT2	3.65	1.09				.46	.71	.53
CONT3	4.29	.75				.65	.86	.81
CONT4	4.60	.62				.53	.78	.70
Futurity	4.03	.61	.76	.40	.76			
FUT1	3.58	.98				.44	.63	.48
FUT2	4.24	.81				.58	.75	.57
FUT3	4.10	.86				.51	.72	.61
FUT4	4.06	.83				.51	.71	.68
FUT5	4.18	.78				.61	.79	.77
Perseverance	4.19	.65	.83	.62	.83			
PER1	4.25	.69				.64	.83	.72
PER2	3.99	.82				.73	.89	.84
PER3	4.32	.74				.70	.87	.80
Organizational identification	4.30	.54	.71	.45	.71			
OI1	4.54	.65				.52	.78	.70
OI2	4.40	.72				.55	.81	.62
OI3	3.97	.66				.53	.79	.69
Collectivism	4.18	.64	.81	.62	.83			
COLL1	4.26	.65				.52	.74	.58
COLL2	4.09	.81				.76	.91	.87
COLL3	4.19	.79				.75	.90	.88
Involvement orientation	4.07	.61	.74	.48	.73			
IO1	4.19	.71				.51	.77	.58
IO2	3.89	.82				.61	.84	.67
IO3	4.13	.73				.57	.82	.81
Low power distance	4.09	.67	.69	.43	.69			
LPD1	3.89	.92				.51	.80	.69
LPD2	4.23	.79				.52	.80	.67
LPD3	4.14	.85				.48	.76	.60
Use of personal power	3.87	.54	.44	.36	.55			
UPP1	4.06	.72				.32	.85	.76
UPP2	4.04	.70				.44	.88	.70
UPP3	3.51	.91				.10	.27	.08
Intrinsic motivation	3.62	.71	.90	.76	.90			
IM1	3.54	.80				.79	.91	.85
IM2	3.72	.74				.84	.93	.91
IM3	3.61	.79				.80	.91	.86
Commitment to learning	4.14	.72	.87	.63	.87			
CTL1	4.22	.81				.76	.88	.88
CTL2	4.12	.83				.75	.87	.89
CTL3	4.13	.86				.67	.81	.67
CTL4	4.10	.87				.73	.85	.72
Shared vision	3.59	.70	.86	.61	.86			
SV1	3.80	.80				.66	.81	.74
SV2	3.55	.87				.74	.86	.82
SV2	3.59	.79				.73	.86	.79
SV4	3.41	.87				.69	.83	.76
Open-mindedness	3.63	.64	.54	.38	.59			
OM1	4.04	.79				.49	.86	.75
OM2	3.62	.88				.40	.83	.74
OM3	3.23	.99				.19	.46	.17
Willingness to change	3.86	.67	.77	.55	.82			
WTC1	4.08	.78				.66	.86	.82
WTC2	4.06	.79				.80	.91	.92
WTC3	3.83	.84				.58	.79	.68
WTC4	3.49	1.03				.39	.58	.43
Error management culture	3.73	.51	.86	.33	.86			
EMC1	3.69	.91				.63	.75	.74
EMC2	3.77	.93				.65	.76	.75
EMC3	3.53	.93				.53	.67	.66
EMC4	3.83	.77				.49	.60	.54
EMC5	4.13	.81				.55	.66	.63
EMC6	4.32	.70				.51	.63	.59
EMC7	3.84	.89				.59	.72	.68
EMC8	3.92	.79				.59	.71	.67
EMC9	4.25	.74				.62	.72	.68
EMC10	3.42	.93				.65	.77	.75
EMC11	3.10	.90				.43	.40	.30
EMC12	3.54	.93				.29	.22	.13
EMC13	3.31	.94				.27	.21	.13
EMC14	3.66	.97				.32	.30	.19
EMC15	3.62	.92				.54	.57	.51
Family commitment culture	4.53	.60	.91	.57	.93			
FCC1	4.82	.54				.73	.81	.79
FCC2	4.57	.81				.58	.67	.63
FCC3	4.64	.63				.74	.81	.79
FCC4	4.74	.65				.79	.85	.84
FCC5	4.30	.90				.71	.76	.73
FCC6	4.58	.71				.80	.85	.84
FCC7	4.56	.68				.73	.79	.77
FCC8	4.37	.84				.69	.75	.72
FCC9	4.46	.88				.79	.83	.80
FCC10	4.26	1.14				.56	.62	.57

*n* = 208; SD: standard deviation, Cr. α: Cronbach's Alpha, AVE: average variance extracted, CR: composite reliability, EFA: exploratory factor analysis, CFA: confirmatory factor analysis.

## 2 Experimental design, materials, and methods

### 2.1 Experimental design

Whenever possible, the measures contained in the provided dataset were borrowed from prior research. When measures and items for a construct were not available, the items were conceptually derived from profound theoretical conceptualizations. To minimize bias in the responses, the questionnaire included different question formats and scale anchors. Further, it contained reverse coded items to minimize acquiescence bias.

Entrepreneurial orientation (EO) was measured as a reflection of five dimensions, namely innovativeness, proactiveness, risk taking, autonomy, and competitive aggressiveness. All items reflecting the individual dimensions were adopted from Covin and Slevin [3], Lumpkin and Dess [4], and Knight [5]. Items for innovativeness, proactiveness, risk taking, and competitive aggressiveness were measured using 5-point semantic differential type scale. For autonomy items, researchers employed a 5-point Likert scale ranging from 1 “not at all” and 5 “to an extreme extent”.

Family commitment culture (FCC) was measured by adopting the family culture dimension of the F-PEC Scale of family influence [6], [7] which is based on the family business commitment questionnaire [8], [9], thereby however removing two items of the original scale because of their focus on the individual rather than the family level of analysis. Researchers employed a 5-point Likert scale ranging from 1 “not at all” and 5 “to an extreme extent”.

The operationalization of long-term orientation (LTO) was inspired by the conceptualization of Brigham et al. [10]. Researchers measured LTO as a second-order construct reflected by three dimensions: futurity, continuity, and perseverance. For all items reflecting those dimensions, a 5-point Likert scale ranging from 1 “not at all” and 5 “to an extreme extent” was employed. While the items for continuity and perseverance are entirely new conceptualizations, four of the five futurity items were adopted from Hoffmann et al. [11] based on the work of Covin and Slevin [3].

The stewardship climate (SCL) concept is based on the conceptualization of Neubaum et al. [12] and was measured as a second-order construct reflected by six dimensions: organizational identification, collectivist orientation, low power distance, involvement orientation, use of personal power, and intrinsic motivation. Again, for all items reflecting those dimensions, a 5-point Likert scale ranging from 1 “not at all” and 5 “to an extreme extent” was employed.

For the concept of learning orientation (LO), researchers relied on Sinkula et al. [13] and employed three dimensions: Commitment to learning, shared vision, and open mindedness. Again, for all items reflecting those dimensions, a 5-point Likert scale ranging from 1 “not at all” and 5 “to an extreme extent” was utilized.

Finally, to consider an organizational culture orientation toward change and tolerance for failure, the data includes measures on willingness to change [14] and error management culture [15]. Again, for all items reflecting those dimensions, researchers employed a 5-point Likert scale ranging from 1 “not at all” and 5 “to an extreme extent”.

### 2.2 Materials

Survey data was collected via an anonymous self-administered web-based questionnaire addressing the owners and/or CEOs of German family firms. Data is available either as comma-separated values (CSV) (.csv) or in the statistical data format provided by the Statistical Package for the Social Sciences (SPSS) (.sav). Furthermore, a Word file contains the full phrasing of the survey items. All files can be accessed via Mendeley Data.

### 2.3 Methods

Relying on the family firm definition provided above, researchers used the Orbis database maintained by Burau van Dijk to identify German family firms. To arrive at the final target population, the following inclusion criteria apply:

• Only firms headquartered in Germany were selected.
• Only mature firms, that is, firms that were founded before 1994 and are at least 25 years old, have at least 25 employees, and a revenue of at least € 5 million were considered for data collection.
• Only those firms in which shareholders are one or more private persons or a family known by name, and in which a shareholder is also a manager, were selected.

The remaining 3,997 firms were cross-referenced with various published directories and individual company websites to ensure the accuracy of the data and identify email addresses. Due to incorrect addresses, firm failures, or firm policies against completing mail surveys, researchers were forced to delete 442 firms from the list which resulted in a final target sample of 3,555 firms. Data collection took place between December 2017 and February 2018 and relied on the key informant approach at the top management level of analysis. An invitation and a link to a web-based survey were sent by email to the owners and/or CEOs of the firms identified. To mitigate ethical concerns in survey research, we aimed at protecting research participants by providing full transparency on the purpose and motivation of the research and ensuring the anonymity of research participants during and after completing the survey (i.e., at no time can conclusions be drawn about the participants or individual statements). After several reminders, the study yielded 404 responses for an initial response rate of 11.4%. However, of those, researchers eliminated responses with missing data. Furthermore, as the questions are related to the perceived strategic focus and culture of the firm, only questionnaires completed by a person in an ownership or top management position were included in the study sample. Moreover, the respondents were asked to classify themselves as being a family business, thereby using two questions: (1) „Are ownership and management control of the company dominated by one family?” and (2) “Do you consider your business to be a family business?”. This procedure yielded a final sample of 208 useful responses for an effective response rate of 5.9%.

## Footnotes

1 The survey instrument includes reverse coded items. However, in the data files (both .csv and .sav), these items have already been recoded by the researchers.
