# Supplementary material for: Survey data on organizational culture and entrepreneurial orientation in German family firms
Source: Data Brief. 2019 Mar 28;24:103827. doi: 10.1016/j.dib.2019.103827 (PMC6463935; doi:10.1016/j.dib.2019.103827)
Supplement: Supplementary file 2 [file mmc2.docx]

**Measures**

| **Entrepreneurial orientation** | | | |
| --- | --- | --- | --- |
| Dimensions | Field(s) | Item(s) | |
| Innovativeness |  | *In general, the top managers of my firm favor . .* | |
|  | INN1 | ...A strong emphasis on the marketing of tried and true products or services | ...A strong emphasis on R&D, technological leadership, and innovations |
|  |  | *How many new lines of products or services has your firm marketed in the past 5 years?* | |
|  | INN2 | No new lines of products or services | Very many new lines of products or  Services |
|  | INN3 | Changes in product or service lines have been mostly of a minor nature | Changes in product or service lines have usually been quite dramatic |
| Proactiveness |  | *In dealing with its competitors, my firm . . .* | |
|  | PRO1 | … typically responds to actions which competitors initiate | … typically initiates actions which competitors then respond to |
|  | PRO2 | ... Is very seldom the first business to introduce new products/services, administrative techniques, operating techniques etc. | ... Is very often the first business to introduce new products/services, administrative techniques, operating techniques etc. |
|  |  | *In general, the top managers of my firm have . . .* | |
|  | PRO3 | ... A strong tendency to “follow the leader” in introducing new products or ideas | ... A strong tendency to be ahead of other competitors in introducing novel ideas or products |
| Risk taking |  | *In general, the top managers of my firm have . . .* | |
|  | RIS1 | ... A strong proclivity for low-risk projects (with normal and certain rates of return) | ... A strong proclivity for high-risk projects (with chances of very high returns) |
|  |  | *In general, the top managers of my firm believe that ...* | |
|  | RIS2 | ... Owing to the nature of the environment, it is best to explore it gradually via careful, incremental behavior | ... Owing to the nature of the environment, bold, wide-ranging acts are necessary to achieve the firm's objectives |
|  |  | *When confronted with decision-making situations involving uncertainty, my firm. . .* | |
|  | RIS3 | ... Typically adopts a cautious, 'wait-and-see' posture in order to minimize the probability of making costly decisions | ... Typically adopts a bold, aggressive posture in order to maximize the probability of exploiting potential opportunities |
| Autonomy | AUT1 | Employees are permitted to act and think without interference | |
|  | AUT2 | Employees perform jobs that allow them to make and instigate changes in the way they perform their work tasks | |
|  | AUT3 | Employees are given freedom and independence to decide on their own how to go about doing their work | |
|  | AUT4 | Employees are given freedom to communicate without interference | |
|  | AUT5 | Employees are given authority and responsibility to act alone if they think it to be in the best interests of the business | |
|  | AUT6 | Employees have access to all vital information | |
| Competitive Aggressiveness |  | *In dealing with its competitors, my firm…* | |
|  | COMP1_r | … is very aggressive and intensely competitive *(reverse coded)* | … makes no special effort to take business from the competition |
|  | COMP2 | ... Typically seeks to avoid competitive clashes, preferring a 'live-and-let-live' posture | ... Typically adopts a very competitive, 'undo- the-competitors' posture |
|  |  |  | |

| **Long-term orientation** | | |
| --- | --- | --- |
| Dimensions | Field(s) | Item(s) |
|  |  | *To what extent do you agree with the following (1=not at all; 5= to an extreme extent)?* |
| Continuity | CONT1 | The management in our firm values decisions and actions that are long lasting. |
|  | CONT2 | The management in our firm values a strong link to the past/ the firm’s history. |
|  | CONT3 | The management in our firm values constancy to pursue an enduring mission. |
|  | CONT4 | Preserving reputations for the longevity of the business is important to our management. |
| Futurity | FUT1 | Planning, forecasting and evaluating the long-range consequences is valuable for the firm. |
|  | FUT2 | The management in our firm focuses in particular on long-term profitability. |
|  | FUT3 | Long-term goals have priority over short-term goals among our management. |
|  | FUT4 | The management in our firm invests deeply into the long-term development of employees. |
|  | FUT5 | The management in our firm emphasizes long-term investments. |
| Perseverance | PER1 | The management in our firm beliefs that efforts made today will be valuable in the future. |
|  | PER2 | The management in our firm demonstrates patience for future rewards. |
|  | PER3 | Persistence is important to our management. |

| **Stewardship climate** | | |
| --- | --- | --- |
| Dimensions | Field(s) | Item(s) |
| Organizational identification |  | *To what extent do the following statements reflect the beliefs of the employees of your company?* |
|  | OI1 | The company’s successes are the employees’ successes. |
|  | OI2 | When someone praises the company, it feels like a personal compliment. |
|  | OI3 | Employees feel a sense of “ownership” for this organization rather than just being an employee. |
| Collectivist orientation |  | *To what extent do the following statements reflect the beliefs of employees in your company?* |
|  | COLL1 | Cooperation among team members usually helps solve problems. |
|  | COLL2 | Team-based work provides the best work performance. |
|  | COLL3 | Teamwork is central to an effective organization. |
| Power distance *(reverse coded)* |  | *To what extent do you agree with the following statements about managerial decision-making*  *behaviors in your company?* |
|  | PD1_r | Managers make most decisions without consulting subordinates. |
|  | PD2_r | Managers frequently use authority and power when dealing with subordinates. |
|  | PD3_r | Managers do not delegate important tasks to employees. |
| Involvement orientation |  | *To what extent do the following statements reflect the beliefs of employees in your company?* |
|  | IO1 | Managers’ decisions are influenced by employees’ input. |
|  | IO2 | Managers try to reach consensus among employees on important decisions. |
|  | IO3 | Managers make employees feel like they work with them, not for them. |
| Use of personal power |  | *To what extent do the following statements reflect the supervisors in your company?* |
|  | UPP1 | Supervisors are individuals that employees can identify with. |
|  | UPP2 | Supervisors give good reasons for changing how employees do their jobs. |
|  | UPP3 | Supervisors have more technical knowledge. |
| Intrinsic motivation |  | *To what extent are employees in your organization satisfied with various facets of their job?* |
|  | IM1 | The extent that supervisors express appreciation to subordinates. |
|  | IM2 | The extent that supervisors give credit to subordinates for their work. |
|  | IM3 | The extent that supervisors give praise to employees for good job performance. |

| **Learning orientation** | | |
| --- | --- | --- |
| Dimensions | Field(s) | Item(s) |
| Commitment to learning |  | *To what extent do you agree with the following (1=not at all; 5= to an extreme extent)?* |
|  | CTL1 | Managers basically agree that our organization’s ability to learn is the key to our competitive advantage |
|  | CTL2 | The basic values of this organization include learning as a key to improvement |
|  | CTL3 | The sense around here is that employee learning is an investment, not an expense |
|  | CTL4 | Learning in my organization is seen as a key commodity necessary to guarantee organizational survival |
| Shared vision | SV1 | There is a commonality of purpose in my organization |
|  | SV2 | There is a total agreement on our organizational vision across all levels, functions and divisions |
|  | SV3 | All employees are committed to the goals of this organization |
|  | SV4 | Employees view themselves as partners in charting the direction of the organization |
| Open-mindedness | OM1 | We are not afraid to reflect critically on the shared assumptions we have made about our customers |
|  | OM2 | Personnel in this enterprise realize that the way they perceive the marketplace must be continually questioned |
|  | OM3_r | We rarely collectively question our own biases about the way we interpret customer information *(reverse-coded item)* |

| **Change orientation** | | |
| --- | --- | --- |
| Dimensions | Field(s) | Item(s) |
| Willingness to change |  | *To what extent do you agree with the following (1=not at all; 5= to an extreme extent)?* |
|  | WTC1 | Managers are generally ready to take on any new challenges that our family firm faces. |
|  | WTC2 | Managers are generally open to trying new things for our family firm. |
|  | WTC3 | Managers are generally fascinated by novel ideas. |
|  | WTC4_r | Managers generally find it hard to change *(reverse coded)* |

| **Error management culture** | | |
| --- | --- | --- |
| Dimensions | Field(s) | Item(s) |
| Error management culture |  | *To what extent do you agree with the following (1=not at all; 5= to an extreme extent)?* |
|  | EMC1 | After an error, people think through how to correct it. |
|  | EMC2 | After an error has occurred, it is analyzed thoroughly. |
|  | EMC3 | In this organization, people think a lot about how an error could have been avoided. |
|  | EMC4 | When an error has occurred, we usually know how to rectify it. |
|  | EMC5 | When an error is made, it is corrected right away. |
|  | EMC6 | Although we make mistakes, we don’t let go of the final goal. |
|  | EMC7 | When people are unable to correct an error by themselves, they turn to their colleagues. |
|  | EMC8 | If people are unable to continue their work after an error, they can rely on others. |
|  | EMC9 | When people make an error, they can ask others for advice on how to continue. |
|  | EMC10 | When someone makes an error, (s)he shares it with others so that they don’t make the same mistake. |
|  | EMC11_r | In this organization, people feel stressed when making mistakes. *(reverse coded)* |
|  | EMC12_r | In general, people in this organization feel embarrassed after making a mistake. *(reverse coded)* |
|  | EMC13_r | In this organization, people get upset and irritated if an error occurs. *(reverse coded)* |
|  | EMC14_r | During their work, people are often concerned that errors might occur. *(reverse coded)* |
|  | EMC15_r | People prefer to keep errors to themselves. *(reverse coded)* |

| **Family commitment culture** | | |
| --- | --- | --- |
|  | Field(s) | Item(s) |
| Family commitment culture  *(originally part of family influence on power, experience and culture (F-PEC) scale)* |  | *To what extent do you agree with the following (1=not at all; 5= to an extreme extent)?* |
|  | FCC1 | We feel loyalty to the family business. |
|  | FCC2 | We are proud to tell others that we are part of the family business. |
|  | FCC3 | We agree with the family business goals, plans, and policies. |
|  | FCC4 | We really care about the fate of the family business. |
|  | FCC5 | There is so much to be gained by participating with the family business on a long-term basis. |
|  | FCC6 | We support the family business in discussion with friends, employees, and other family members. |
|  | FCC7 | Your family and business share similar values. |
|  | FCC8 | Your family members share similar values. |
|  | FCC9 | Our family members are willing to put in a great deal of effort beyond that normally expected  to help the family business be successful. |
|  | FCC10 | Your family has influence on your business. |

*Controls*

|  | Field(s) | Item(s) |
| --- | --- | --- |
| Confirmation of family business | FB1 | Are ownership and management control of the company dominated by one family?  1=*yes; 2=no* |
|  | FB2 | Do you consider your firm a family business?  1=*yes; 2=no* |
| Generational involvement | GI | How many generations are currently involved in your family firm?  *1=One generation, 2=two generations, 3=multiple generations (more than two)* |
| Involvement of the founder | IF | Is the founder of this firm still involved in the management of the company?  1=*yes; 2=no* |
| Role | Control role | *1=owner, 2=owner and CEO, 3=CEO, 4=employee in leadership position, 5=employee* |
| CEO tenure | Control_ceo tenure | Worked in the family firm since: ____ (fill in) |
| Industry | Control_industry | *1=Automotive, 2=Real Estate, 3=Bio/ Medical Technology, 4=Electronics Industry, 5=Chemicals/ Pharmaceuticals, 6=Energy/ Resources, 7= Financial Services, 8=Trade, 9=IT/Software/Internet, 10=Engineering, 11=Media, 12=Professional Services, 13=Telecommunications, 14=Transport/Logistics, 19=Others* |
| Firm age | Control_age | Founded in: ____ (fill in) |
| Firm size | Control_size | Total number of employees relative to competitors:  *1 = bottom 20%, 2 = next lowest 20%, 3 = middle 20%, 4 = next highest 20%, and 5 = top 20%* |
| Prior firm performance | Control_prior performance1 | Total sales growth over the most recent year compared to industry competitors:  *1 = bottom 20%, 2 = next lowest 20%, 3 = middle 20%, 4 = next highest 20%, and 5 = top 20%* |
|  | Control_prior performance2 | After-tax return on sales over the most recent year compared to industry competitors:  *1 = bottom 20%, 2 = next lowest 20%, 3 = middle 20%, 4 = next highest 20%, and 5 = top 20%* |
